# Fortified Snack Reduced Anemia in Rural School-Aged Children of Haiti: A Cluster-Randomized, Controlled Trial

**DOI:** 10.1371/journal.pone.0168121

**Published:** 2016-12-22

**Authors:** Lora Iannotti, Sherlie Jean-Louis Dulience, Saminetha Joseph, Charmayne Cooley, Teresa Tufte, Katherine Cox, Jacob Eaton, Jacques Raymond Delnatus, Patricia B. Wolff

**Affiliations:** 1 Institute for Public Health, Brown School, Washington University in St. Louis, St. Louis, Missouri, United States of America; 2 Meds & Food for Kids, St. Louis, Missouri, United States of America; National Cancer Institute, UNITED STATES

## Abstract

**Background:**

Nutrition in the school-aged child matters for brain development and public policy investments globally. Our group previously conducted a trial in urban schools of Haiti to examine the effects of a fortified peanut butter snack, Vita Mamba, with limited findings for anemia.

**Objective:**

We aimed to test the hypothesis that Vita Mamba, with systematic deworming in both study arms, would significantly reduce anemia among rural, school-aged children.

**Methods:**

A cluster, randomized longitudinal study was conducted in two rural communities of the North-East Department of Haiti, 2014–2015. Healthy children ages 3–16 years were enrolled (*n* = 321) and assigned by school to intervention (Vita Mamba and deworming) and control (deworming). Vita Mamba contains 260 kcal and meets >75% of the Recommended Dietary Allowance for critical micronutrients. Multivariate regression analyses including propensity score matching techniques to correct for potential group imbalance (Kernel-based Matching and Propensity Score Weighting) were applied to examine difference-in-difference intervention effects.

**Results:**

At baseline, 51% of the children were anemic with no significant differences between study groups. Vita Mamba supplementation showed a consistent, positive effect across regression models on increasing Hb concentration and reducing the odds of anemia compared to the control group after adjusting for child age, vitamin A supplementation, milk consumption, and height-for-age *z* score. The average treatment effect for the treated in the Propensity Score Weighting models was 0.62±0.27 grams per 100 milliliters (g/dL) for Hb concentration (F = 4.64, *P* = 0.001), and the odds of anemia were reduced by 88% (Wald ***χ*²** = 9.77, *P* = 0.02). No differences in change in anthropometric markers were evident.

**Conclusions:**

School feeding programs that integrate fortified foods with deworming could reduce anemia burden with important implications for learning, health, and well-being. The rural-urban differences in anemia require further study.

## Introduction

Nutrition in the school-aged child has been an underrepresented public health problem in recent years, overshadowed by the emphasis on nutrition in the first 1,000 days. From both physiological and policy perspectives, there are compelling reasons to also focus on nutrition in this age group. Child development processes are ongoing during the school-age period, with particularly rapid growth evident in the brain’s prefrontal cortex and hippocampus [1]. Nutrition insults in the school-aged child may impair learning, increase susceptibility to infection, and negatively impact reproductive health over the long-term [2]. School feeding programs represent the largest public investment in food programs globally, yet evidence is minimal for nutrition impacts [3,4]. With appropriately designed and targeted nutrition interventions, schools could be further leveraged as ideal platforms for improving health and development in societies.

Anemia arises from multiple etiologies including undernutrition and infection, but is too often addressed through interventions that focus on single causes such as iron supplementation or deworming. Our previous trial in six urban schools of Cap-Haitien aimed to test the effects of a fortified peanut butter snack, Vita Mamba (then called Mamba), on anemia and other nutrition outcomes [5]. Vita Mamba contains 260 kcal and meets >75% of the Recommended Dietary Allowance (RDA) for critical micronutrients for children in the age range, 4–8 years. Vita Mamba significantly increased body mass index (BMI), fat mass, and proportion of fat mass among the school-aged children, with thinness highly prevalent at baseline. The results for anemia and changes in hemoglobin (Hb) concentration, however, were limited. Vita Mamba reduced the odds of children developing anemia by only 28%, and no treatment effect was found for Hb concentration. In subsequent analyses, we showed the highly prevalent anemia in the school children (70.6%) was associated with stunting, fever, poultry ownership, and absence of deworming and vitamin A supplementation [6].

This evidence prompted refinements in the original hypotheses and a new study in a rural area of Haiti. Based on the previous results, anemia in the school children was likely arising from multiple causes beyond nutrition in this context, and it was reasoned that if helminth infection in particular was controlled, a significant impact might be realized from Vita Mamba. Deworming in schools in Haiti is the national protocol, but was not systematically followed in the urban trial. This study aimed to test whether Vita Mamba supplementation would significantly reduce anemia and increase Hb concentrations, with deworming of all participating school children. The aim was achieved as evidenced by our findings for significant increases in Hb concentration and reduced odds of anemia.

## Methods

### Study schools and participants

**Fig 1:**
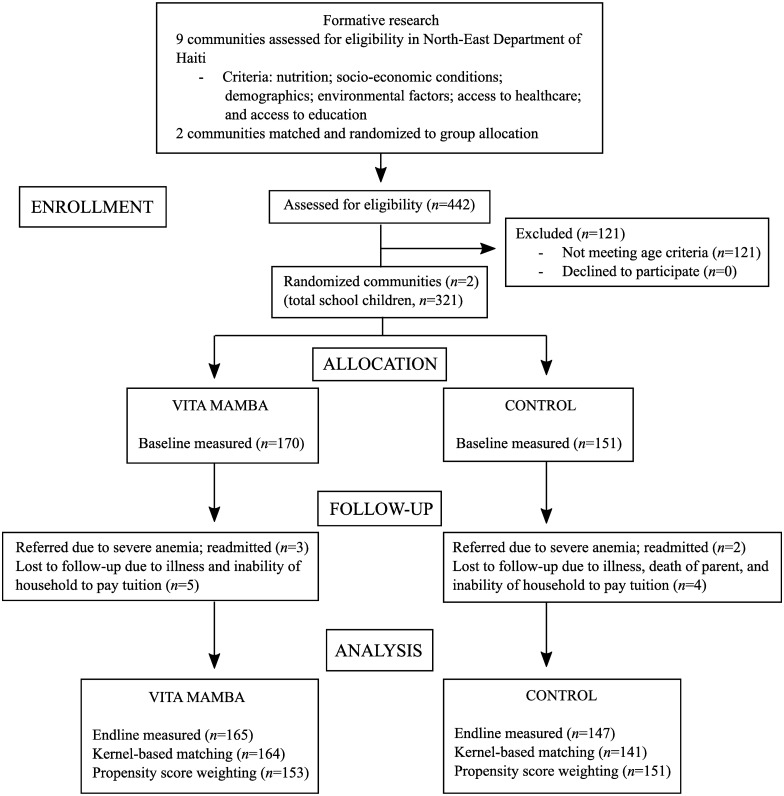
Flow diagram of Vita Mamba trial in Haitian school children. Formative research identified eligible communities based on a set of criteria. The two selected communities were randomly assigned to one of the two study arms. After parents were informed, eligible children were recruited and enrolled. Parents were surveyed on socio-economic and demographic characteristics, child diet, and child morbidities at baseline, and children were followed at two time points, baseline and endline, for measures of Hb concentration, height, and weight. Data was analyzed for intervention effects using regression modeling including kernel-based matching (KBM) and propensity score weighting (PSW).

Formative research was carried out for a period of 2 months to identify and match rural communities for inclusion in this study (Fig 1). Our objective for this study was to examine the effects of Vita Mamba in a representative rural setting of Haiti. The search was focused in rural areas in the North-East Department of Haiti with relative proximity to Cap-Haitien and our research infrastructure. Prevalence of stunting among children less than five years in the North-East (22.1%) exceeds but is comparable to the national average (21.9%) [7]. We explored 9 communities (Carice, Mont-Organise, Terrier-Rouge, Grand-Bassin, Jacquesil, Derac, Dolval, and Danda) in the region where there was feasible access from Cap-Haitien and existing relationships with communities. The following criteria were used to both identify comparable communities for internal validity, and representativeness as a Haitian rural context for external validity: nutrition (stunting prevalence, typical diet); socioeconomic (sources of livelihood, rain-fed agriculture, access to credit, market location); demographic (population of entire community, and specifically, school-aged children), environmental characteristics (water sources, rainfall patterns); access to healthcare (nearest clinic and hospital, presence of community health agents); and access to education (preschool, primary school, and high school). Communities were excluded if they had an existing school feeding program. Two communities, similar across these characteristics were ultimately selected: Dolval, with an approximate population of 6,500; and Danda, with an approximate population of 8,000. All 3 primary schools in the 2 communities were included (2 schools in Dolval and 1 school in Danda), with each community representing a cluster. Random assignment was carried out through allocation-concealment using sealed paper forms containing the group assignment, finally designated as treatment (Dolval, 2 schools) and control (Danda, 1 school).

### Recruitment and enrollment

All parents from the 3 primary schools in the communities were contacted through phone calls and parent meetings held at the school prior to the start of the academic year, during the months of August and September, 2014, to provide information about the student. The recruitment and enrollment period was from November 3, 2014, until December 1, 2014. All parents or caregivers from the three schools expressed an interest in participating in the study. Children were screened for eligibility based on the following criteria: registration in the study school for 2014–2015 calendar; good health (no fever, congenital health condition, or peanut or soy allergy); age (3–16 years); and not severely malnourished (weight-for-height [WHZ] <-3). Due to relatively low population density compared to the urban study site and sample size needs, we expanded the age range criteria from what is typically considered school-age. To account for potential differences by age, we stratified analyses and adjusted for child age in regression modeling.

Parents of eligible children went through the informed consent procedure and children were asked to give verbal assent. In the case of illiteracy, written consent forms were read aloud to parents and signatures or crosses obtained to signify consent. Of the total 442 students recruited from the two communities, 321 children were considered eligible: 121 students were excluded due to age criteria (either less than 3 years or older than 16 years); and no parents of eligible children declined to participate (Fig 1).

Power calculations were used to determine the adequacy of sample size, predetermined by total number of eligible children in the study schools and study budget. Using an estimate of difference-in-difference, mean change in Hb of 0.40 (SD 1.2) based on previous studies, adequate sample size need was estimated to be 143 per group [alpha = 0.05 and power (1-β = 0.80)] [8]. With a design effect of 1.06 applied and estimated losses-to-follow-up of 10%, the study was found to be adequately powered to detect the hypothesized difference in Hb with 321 children enrolled [9].

The study including the consent procedure as previously described was approved by the National Bioethics Committee of the Ministry of Health (Ministère de la Sante Publique et de la Population, MSPP) in Haiti on October 20, 2014 and the Institutional Review Board of the Human Research Protection Office of Washington University in St. Louis on October 9, 2014. The study was registered at ClinicalTrials.gov as NCT02747524 (https://clinicaltrials.gov/ct2/show/NCT02747524). Registration was completed after enrollment of participants and the trial was finished, because it had not been a requirement by the funding sources. The authors confirm that all ongoing and related trials for this intervention are registered.

### Study design and intervention

This study applied a matched cluster, controlled design. Children were followed longitudinally for Hb concentration and anthropometric measures were taken at baseline (November/December 2014) and endline (May/June 2015). The entire range of follow-up was from November 20, 2014 until June 22, 2105. Parents were surveyed at baseline for household-level socio-economic and demographic information; environmental conditions including water, hygiene, and sanitation; and child diet and morbidities.

All children in the intervention schools received the Vita Mamba once per school day from November 2014 to June 2015, for approximately 26 weeks. Children in both groups received albendazole at baseline in November 2014. Vita Mamba is a fortified peanut butter paste that can be consumed directly from the package. Students in the intervention schools were first given hand sanitizer to clean hands prior to receiving the Vita Mamba. Next, teachers opened the sealed package of Vita Mamba and distributed one to each student. This was carried out to discourage selling or sharing the food with family members after school. Compliance was monitored by teachers and tracked by collection of empty packages. At the close of snack time, empty packages were collected, counted, and returned to the factory for proper disposal.

Vita Mamba was developed for the urban school trial by our research team with representatives from Edesia (a US-based nonprofit manufacturer of ready-to-use foods with expertise in research and development) and Nutriset (the parent company in the PlumpyField network). Meds & Food for Kids, a nonprofit manufacturer of ready-to-use foods in Haiti using locally produced peanuts, produced the Vita Mamba for this trial. Vita Mamba (50 g) contains 260 kcal and meets >75% of the RDA for critical micronutrients for children ages 4–8 years (S1 Table). We designed the food based on the RDA of this age group primarily because there was the highest proportion of the sample of school-aged children falling into this age range, also a critical period requiring nutrients for growth and development. As well, if designed for older children, nutrient intakes for younger children may have exceeded needs with potential negative consequences.

### Nutrition and health parameters

Hb concentrations were measured using the Hemocue system and reported in grams per 100 milliliters (g/dL). The Hemocue system was cleaned and calibrated each morning before sample collection. Blood was collected with a fingerstick and a microcuvette in one continuous process and tested in the Hemocue system. Results were compared to World Health Organization (WHO) anemia cut-offs for anemia: 0–4.9 years, 11.0 g/dL; 5–11.9 years, 11.5 g/dL; 12–14.9 years, 12.0 g/dL; non-pregnant girls 15–16 years, 12.0 g/dL; boys 15–16 years, 13 g/dL [10].

For anthropometry, the Seca Model 874 (Digital) 440 lb x 0.1 lb resolution scale and the ShorrBoard height measuring board were used to collect child weight (to the nearest 0.1 kg) and height measures (to the nearest 1 mm), respectively, using international protocols [11]. The study team took two measures of child length and weight, and if there was a difference greater than 0.5 cm or 0.5 kg respectively, a third measure was taken. Height and weight measures were used to calculate BMI [weight (kg)/height (m)^2^], anthropometric z-scores, and the prevalence of stunting [height-for-age *z* score (HAZ)<-2], underweight [weight-for-age *z* score (WAZ)<-2], wasting [weight-for-height *z* score (WHZ)<-2], and thinness [BMI *z* score (BMIz)<-2] based on WHO Growth Standards (2006) for children 3–5 years and WHO Growth References (2007) for children 6–13 years [12,13]. Children identified during the study with severe acute malnutrition (SAM) (WHZ<-3) or severe anemia (Hb< 7 g/dL) were removed from the study and referred to the healthcare system. Five children were referred for severe anemia and treated for infection during the follow-up period, 3 from the control group and 2 from the intervention group. All 5 children were re-admitted to the study after recovery defined as Hb>7 g/dL.

Child diet was assessed using a 24-hour food frequency of intake administered during the parent survey at baseline. There were 17 foods included as those commonly consumed by this age group in Haiti. The foods were identified during the formative research phase as those that school children may access and might consume. A previously validated dietary diversity score, ranging from 1–17, was calculated by summing the total number of different food items consumed in a 24-hour period [14]. Child morbidity, also collected at baseline, was assessed using a two-week period of recall for diarrhea [acute diarrhea (three or more semi-solid or liquid stools in a 24-hour period), number of days with acute diarrhea, and bloody diarrhea]; and during the previous month for all other morbidities [malaria, fever, respiratory condition (cough plus short, rapid breathing), eye infection, ear infection, skin conditions, and helminth infection]. Caregivers were also asked to report whether the child or any family members had a history of peanut or soy allergies.

### Statistics

Comparability of study groups on baseline child nutrition and household-level socioeconomic, demographic, and environmental factors were first examined by univariate statistics, chi-squared, *t*-tests, and ANOVA. Next, we examined primary outcomes (changes in Hb concentrations and anemia status) and secondary outcomes (changes in anthropometry and undernutrition status), using univariate methods. Values for continuous outcomes are presented as means ± SD, except for coefficient values in regression models presented as means ± SEE. *P* values < 0.05 were considered significant.

The third phase of statistical analyses applied multivariate regression modeling. Simple regression techniques were first applied using Ordinary Least Squares (OLS) modeling for continuous outcomes and logistic regression for dichotomous outcomes. To further correct for any potential selection bias in the cluster design, we applied established propensity score matching (PSM) methods during the analysis phase [15]. Different PSM techniques were used to compare effect findings across models, and we selected those ultimately that minimized sample losses. We tested all variables hypothesized to potentially confound the effects across models, and retained the covariates reaching significance or trending significance p<0.10.

Kernel-based matching (KBM) uses propensity scores differentially to calculate a weighted mean of counterfactuals, constructing matches from all individuals in the control group without significant loss of sample size [16,17]. This method derives the average treatment effect for the treated (ATT), using one-to-many matching with lowess or local linear regression calculation, weighting information from closer matches and down-weighting those further from the treatment participants. The differences in Hb concentration for the treated cases, the children receiving Vita Mamba through the academic year, were compared to the weighted average differences in Hb concentration outcomes of the non-treated (control) cases. Reported results use the default bandwidth of 0.06; sensitivity analyses with varying bandwidths and trimming regions showed a consistent positive effect.

Propensity score weighting (PSW) applies the inverse probability of treatment assignment as a weight in a multivariate analysis, enabling the model to use varying degrees of information from each participant depending on their conditional probability of receiving the treatment [15,18]. Two types of weighting estimators were calculated and reported from our data: average treatment effect (ATE) and ATT. The ATE model produces results based on intent-to-treat analyses and provides estimates for the treatment effect of Vita Mamba for a randomly selected sample from the population, while the ATT, generally considered of greater substantive interest, estimates the treatment effect only for those who received Vita Mamba supplementation [15,19]. Data analyses were performed with STATA software (version 13.1; StataCorp, College Station, TX).

## Results

Over one-half of the children were anemic at baseline (Table 1). Groups did not differ significantly on undernutrition status, with the exception of underweight prevalence. Morbidities potentially related to anemia also did not differ by group, and were infrequently reported by caregivers: 1 child for malaria and 3 children for helminth infection. Dietary diversity was greater among children in the control group, though animal source food intake did not differ by study arm. Livestock ownership was pervasive among households, with no statistical differences evident by study group. Significantly more households in the Vita Mamba group owned land compared to the control group. On average, agriculture was reported to be the primary occupation of caregivers, 37.5% of mothers and 51.3% of fathers.

**Table 1:** Baseline socioeconomic and demographic characteristics of Haitian school-aged children.

		Vita Mamba (*n* = 170)	Control (*n* = 151)	All (*n* = 321)
**Child age,** ^a^^,^^b^ **y**		7.6 ± 2.9	8.6 ± 3.5	8.0 ± 3.2
**Sex of child, %**	**Female**	48.8	46.7	47.8
	**Male**	51.2	53.3	52.2
**Undernutrition Status**	**Stunted, %**	14.8	9.3	12.2
	**Underweight,** ^b^ **%**	15.1	6.8	11.4
	**Thin, %**	11.8	13.3	12.5
	**Anemic, %**	54.1	47.7	51.1
**Hemoglobin concentration,** ^a^ **g/dL**		11.2 ± 1.4	11.4 ± 1.4	11.3± 1.4
**BMI**^a^^,^ ^b^		14.5 ± 1.4	15.1 ± 1.6	14.8 ± 1.5
**Child diet**	**Dietary diversity score**^a^^,^^b^	4.0 ± 1.6	4.8 ± 1.6	4.4 ± 1.6
	**Animal source food intake, %**	35.3	37.1	36.1
**Maternal age,** ^a^ **y**		37.2 ± 12.7	38.7 ± 12.9	37.9 ± 12.8
**Maternal primary education or higher, %**		34.5	28.7	31.8
**Total number of household members**^a^^,^^b^		7.3 ± 2.6	6.7 ± 1.9	7.0 ± 2.3
**Household monthly income ($US),** ^b^ **%**	**10–50**	65.3	47.6	57.0
	**51–80**	10.2	8.8	9.5
	**81–100**	17.3	12.9	15.3
	**101+**	7.2	30.7	18.2
**Land ownership,** ^b^ **%**		71.8	34.7	54.4
**Livestock ownership, %**		82.4	80.0	81.3
**Drinking water source,** ^b^ **%**	**Faucet inside home**	5.9	0.0	3.2
	**Public pump**	63.5	94.0	77.8
	**Spring**	30.6	4.0	18.1
	**River/lake**	0.0	2.0	0.9
**Latrine toilet use,** ^b^ **%**		47.1	82.1	63.6
**Mosquito net ownership,** ^b^ **%**		65.3	52.3	59.2

^a^ Values are mean ± SD.

^b^ Groups significantly different by ANOVA, *t* test, or chi-squared, *P*<0.05.

In the intervention group, there was a higher proportion drawing water from a spring compared to those in the control group where the predominant water source was public pumps. Median time to retrieve water was 5 minutes with no significant difference by group, though 22.4% of households reported time to retrieve water was 15 minutes or greater. No households were reported to be using a flush toilet; households in the control group reported higher latrine use compared to the intervention group. Parent-reported prevalence of child morbidities was low and did not differ by treatment group: fever (6.3%); diarrhea (2.8%); eye infection (7.5%); ear infection (5.9%); and skin conditions (9.7%).

No statistical differences in change in HAZ, WAZ, and BMIz by treatment group were evident (Table 2). Children ages 11 to 16 years showed the greatest reduction in HAZ score from baseline to endline compared to other age groups (ANOVA, *P* = 0.01). Hb concentration increased across all age groups in the Vita Mamba group and decreased by endline for all age groups of the control groups, reaching statistical significance in the children ages 11 to 16 years. Changes in stunting, underweight, and thin status did not differ by treatment group from baseline to endline. However, a significantly greater percentage of children in the control group (27.9%) became anemic by endline compared to the Vita Mamba group (15.8%) (chi-squared, *P* = 0.01).

**Table 2:** Change in anthropometry and hemoglobin concentration of Haitian school-aged children from baseline to endline, by treatment group. ^a^

		Vita Mamba	Control	All
**Height-for-age *z* score**	**3–5 y (*n* = 89)**	0.01 ± 0.20	0.03 ± 0.53	0.02 ± 0.38
	**6–10 y (*n* = 149)**	−0.04 ± 0.15	−0.07 ± 0.12	−0.05 ± 0.13
	**11–16 y (*n* = 83)**	−0.23 ± 0.86	−0.11 ± 0.35	−0.16 ± 0.62
	**All (*n* = 311)**	−0.06 ± 0.43	−0.05 ± 0.35	−0.06 ± 0.39
**Weight-for-age *z* score**	**3–5 y (*n* = 89)**	−0.01 ± 0.23	−0.01 ± 0.27	−0.01 ± 0.24
	**6–10 y (*n* = 149)**	0.12 ± 0.30	0.11 ± 0.22	0.11 ± 0.27
	**All (*n* = 207)**	0.06 ± 0.28	0.06 ± 0.25	0.06 ± 0.27
***BMI z* score**	**3–5 y (*n* = 89)**	−0.03 ± 0.38	−0.05 ± 0.60	−0.04 ± 0.49
	**6–10 y (*n* = 149)**	0.21 ± 0.46	0.22 ± 0.37	0.22 ± 0.42
	**11–16 y (*n* = 83)**	0.36 ± 0.95	0.31 ± 0.36	0.33 ± 0.67
	**All (*n* = 311)**	0.17 ± 0.59	0.18 ± 0.46	0.18 ± 0.53
**Hemoglobin concentration, g/dL**	**3–5 y (*n* = 89)**	0.25 ± 1.35	−0.12 ± 1.38	0.08 ± 1.37
	**6–10 y (*n* = 149)**	0.09 ± 1.03	−0.17 ± 1.25	−0.02 ± 1.13
	**11–16 y (*n* = 83)**^b^	0.12 ± 1.52	−0.76 ± 1.14	−0.39 ± 1.37
	**All (*n* = 312)**	0.14 ± 1.28	−0.34 ± 1.28	−0.09 ± 1.27

^a^ Values are mean ± SD.

^b^ Groups significantly different by *t* test, *P*<0.05.

In regression modeling, Vita Mamba supplementation consistently showed a positive effect on increasing Hb concentration and reducing the odds of anemia compared to the control group after adjusting for child age, vitamin A supplementation, milk consumption, and HAZ (Table 3). Effect size varied by model type. KBM generated an ATT comparable to the OLS model, though with bootstrapping applied to derive the standard error, the bias-corrected 95% CI is no longer significant. All PSW models showed significantly positive effects for increasing Hb concentration and reducing the odds of anemia from Vita Mamba with greater effect sizes than the OLS and KBM models.

**Table 3:** Regression models for change in hemoglobin concentration and anemia status of Haitian school-aged children, by treatment group.

	OLS^a^ (*n* = 312)	Logistic (*n* = 312)	KBM, (*n* = 299)	PSW, ATE (*n* = 176)		PSW, ATT (*n* = 176)	
	Hemoglobin concentration, g/dL	Anemia	Hemoglobin concentration, g/dL	Hemoglobin concentration^b^, g/dL	Anemia^c^	Hemoglobin concentration^b^, g/dL	Anemia^c^
**Mamba ± SE**	0.46 ± 0.14	0.47 ± 0.13	0.46 ± 0.30	0.56 ± 0.21^d^	0.33 ± 0.15^d^	0.62 ± 0.27^d^	0.22 ± 0.12^d^
**Statistic**	F = 10.3	LR ***χ*²** = 7.24	NC^e^	F = 6.32	Wald ***χ*²** = 11.5	F = 4.64	Wald ***χ*²** = 9.77
**Probability**	<0.001^f^	0.001^g^	95% CI = -0.09–0.95^h^	<0.001^e^	0.01^f^	0.001^e^	0.02^f^

Average treatment effect, ATE; average treatment effect for the treated, ATT; kernel-based matching, KBM; ordinary least squares, OLS; propensity score weighting, PSW.

^a^ OLS adjusts for child age.

^b^ PSW hemoglobin adjusts for child age, vitamin A supplementation, and consumption of milk.

^c^ PSW anemia adjusts for height-for-age z score and vitamin A supplementation.

^d^ Robust standard error.

^e^ NC = not comparable due to bootstrap.

^f^ Probability > F.

^g^ Probability > ***χ*²**.

^h^ The current statistical software packages are unable to calculate SE using the lowess function employed by KBM, the bootstrapping SE is used. This provides the ability to estimate a 95% CI but not a specific *P* value.

## Discussion

This school feeding study in a rural area of North-East Haiti showed that supplementation with a fortified snack for six months, with systematic deworming in both trial arms, produced a consistent positive effect on reducing anemia and increasing Hb concentration. At baseline, we found a high prevalence of anemia among all children in this rural site (51%), though relatively lower than the prevalence found in our previous urban school feeding study (70.6%) [6]. After adjusting for group imbalance and further controlling for age, HAZ, vitamin A supplementation, and milk consumption, the ATT in the PSW model showed Vita Mamba increased Hb concentration by 0.62 g/dL and reduced odds of anemia by 88% compared to control. All other regression models showed findings for increased Hb concentration, by 0.46 g/dL for the OLS and KBM models, and 0.56 for ATE of the PSW model, and reduced odds of anemia by 53% for logistic regression and 67% for ATE. No differences in change in anthropometric markers were evident.

Findings from this rural site differed from the urban school feeding research conducted in 2012–2013 in Cap-Haitian schools [5]. Although Vita Mamba (previously called Mamba) minimally reduced the odds of anemia by 28% in the earlier research, there was no significant increase in Hb concentration. We hypothesized that in the urban environment, more densely populated and characterized by poor water and sanitation conditions, anemia-related infectious diseases in the school children could have confounded nutrition impacts of Vita Mamba. Thus by systematically deworming across all children, the nutrition-related anemia effects might become more apparent. In the rural area, we also observed a low prevalence of fever, diarrhea, and other infectious disease morbidities at baseline despite widespread use of open defecation and latrines for sanitation that may have further highlighted the nutrition effects. The relatively sparse population density may be contributing to reduced infection transmission. Neither study found increased HAZ or reduced stunting which was expected given the older age of the children. Other body composition parameters measured using bioelectrical impedance were improved in the urban study, but not investigated in this rural site again due to resources constraints.

Few studies globally have examined anemia disparities in school-aged children according to an urban-rural divide. Studies in Ethiopia and Cameroon similarly found a higher anemia prevalence in primary school children living in urban settings compared to rural, while another in Uganda showed higher anemia in a peri-urban versus rural setting, but with some variations by age [20–22]. There is some evidence from studies in young children for the urban-rural disparities, but findings have been mixed [23,24]. One extensive review of Demographic and Health Surveys data from 47 countries examined nutrition and other health disparities by urban-rural residence, finding overall child health to be better in urban areas [25]. However, once socioeconomic status was accounted for, the urban poor had higher rates of stunting and mortality than rural counterparts. Our trials, both carried out in resource-poor populations, seem to confirm this.

Since our first Vita Mamba trial, there have been some updates in the evidence-base for school feeding interventions that have similarly used fortified food or beverages, many with comparable findings for improved micronutrient nutrition [26–32]. Fortified packaged foods also have the advantage of convenience over cooked foods, which may be important in resource-constrained schools with limited facilities and staff. One study in Ghana testing a micronutrient-fortified corn soya blend found significant increases in Hb concentration, but no effects on anthropometry and other micronutrient biomarker parameters [26]. There was no difference in iron deficiency anemia between groups, suggesting the contribution of other anemia-related micronutrients. Systematic reviews of micronutrient-fortified foods compared to single nutrients or unfortified foods have similarly shown positive results for school-aged children on reductions in anemia and other health outcomes [2,33].

In an analysis from our urban trial, vitamin A supplementation was positively associated with Hb concentration, and here it predicted reduced anemia and Hb outcomes in regression modeling [6]. Other fortified food inventions with vitamin A have also shown effects on reducing anemia [29,34]. Although the role of vitamin A in hematopoiesis is not fully characterized, deficiency is known to contribute to microcytic anemia potentially through iron metabolism or red blood cell differentiation pathways [35,36]. The decrease in the Hb concentration found across all age groups in the control group from baseline to endline may be due to seasonality of foods containing vitamin A or other nutrients related to anemia.

Throughout childhood, there are ongoing growth processes that at a certain age may no longer manifest as rapid changes in weight and height, but may be no less important to the development of the child. Nutrient inadequacies, infection, or other environmental insults may impede these processes and limit potential. Relatively less is understood about nutrition in the school-aged child from poor countries compared to other periods in the life cycle, but evidence suggests nutrition insults can impair cognitive and other domains of development [37–40]. Anemia, arising from any of its multiple etiologies, diminishes oxygen transport in the body and to the brain where metabolic needs are high. In the school-aged child, anemia might interact with other environmental challenges to damage vital socio-emotional and cognitive-language development [41]. The recently released findings for global burden of disease among children and adolescents found iron deficiency anemia to be the leading cause of years lived with disability among both children and adolescents [37].

Our study was limited primarily by the small cluster design. Recognizing this constraint early in the process, however, enabled us to take several precautions to protect internal validity. Formative research was conducted to identify and match communities, though some baseline characteristics differed between the treatment and control groups. We addressed these differences in regression modeling by adjusting for the factors, applying PSM techniques, and using longitudinal difference-in-difference design. These methods produced both consistent findings and a relatively large effect size for Vita Mamba on Hb and anemia that offer credibility to the causal inference. However, we recognize there remains risk for residual confounding. Another potential limitation in the study could be the wide age range of the participating children, 3–16 years. Responsiveness to treatment may have varied by age. To address this, we stratified by age groups based on physiological commonalities in growth and development. Although effect size varied by group, there was a consistent trend for improved Hb concentration. This study, despite its limitations, showed that with treatment with albendazole across both groups, Vita Mamba had a significant impact on reducing anemia and increasing Hb concentration. The next step might be to now conduct a large-scale trial in both urban and rural schools to simultaneously test the integrated Vita Mamba and deworming effects.

Ongoing development processes in the school-aged child through the next 3,000 days of life may be impaired by anemia and related nutrient deficiencies, necessitating thoughtfully designed and targeted interventions. We showed that an integrated approach combining Vita Mamba and deworming had significant impacts on reducing anemia in school children. In our view, these findings combined with those from the urban study justify more comprehensive programs to address anemia in the school-aged child. Programs may vary by context depending on the relative contributions of different factors leading to anemia, but in Haiti, there appears to be a need for both nutrition and infection-related interventions. Further research may also be needed to understand vitamin A-related and other anemia etiologies in the school-aged population of children.

## Supporting Information

S1 Table Nutritional composition of intervention snacks. Recommended Dietary Allowance, RDA.
(DOCX)

S1 Protocol Protocol: Fortified Snack Reduced Anemia in Rural School-Aged Children of Haiti: A Cluster-Randomized, Controlled Trial. (DOCX)

S1 Checklist CONSORT Checklist. (DOCX)
