# Supplementary material for: Fortified Snack Reduced Anemia in Rural School-Aged Children of Haiti: A Cluster-Randomized, Controlled Trial
Source: PLoS One. 2016 Dec 22;11(12):e0168121. doi: 10.1371/journal.pone.0168121 (PMC5179061; doi:10.1371/journal.pone.0168121)
Supplement: S1 Table — Recommended Dietary Allowance, RDA. (DOCX) [file pone.0168121.s001.docx]

| Nutrients | Unit | RDA [11]  (4-8 years) | Vita Mamba (per 50 g sachet) | % RDA met by Vita Mamba |
| --- | --- | --- | --- | --- |
| Energy | kcal | 1800 | 260 | 14.4 |
| Protein | g | 19 | 9.1 | 47.9 |
| Lipids (% of daily energy requirements) | % | 25-35 | 9 | 30 |
| Linoleic Acid (18:2n-6) | g | 10 | 5.4 | 54 |
| α-Linolenc Acid (18:3n-3) | g | 0.9 | 0.7 | 77.8 |
| Docosahexaenoic acid (22:6n-3) | mg | - | 35 | - |
| Carbohydrates | g | 130 | 19 | 14.6 |
| Vitamins |  |  |  |  |
| Vitamin A, RAE | μg | 400 | 305 | 76.3 |
| Thiamin (B1) | mg | 0.6 | 0.82 | 135.8 |
| Riboflavin (B2) | mg | 0.6 | 0.82 | 135.8 |
| Niacin (B3) | mg | 8 | 5.90 | 73.7 |
| Pantothenic acid (B5) | mg | 3 | 0.76 | 25.3 |
| Vitamin B6 | mg | 0.6 | 0.6 | 100 |
| Vitamin B12 | μg | 1.2 | 1.2 | 100 |
| Folate, DFE | μg | 200 | 165 | 82.5 |
| Vitamin C (ascorbic acid) | mg | 25 | 59 | 236 |
| Vitamin D (D2+D3) | μg | 5 | 7.7 | 154 |
| Vitamin E (alpha-tocopherol) | mg | 7 | 4.54 | 64.8 |
| Vitamin K | μg | 55 | 17.55 | 31.9 |
| Minerals |  |  |  |  |
| Calcium | mg | 800 | 190 | 23.8 |
| Copper | mg | 0.44 | 0.45 | 102.3 |
| Iodine | μg | 90 | 140.5 | 156.1 |
| Iron | mg | 10 | 11 | 110 |
| Magnesium | mg | 130 | 75 | 57.7 |
| Manganese | mg | 1.5 | 11 | 733.3 |
| Phosphorus | mg | 500 | 200 | 40 |
| Potassium | mg | 3800 | 493 | 13.0 |
| Selenium | μg | 30 | 23 | 76.7 |
| Sodium | mg | 1200 | 44 | 3.7 |
| Zinc | mg | 5 | 4.9 | 98 |
