# Supplementary material for: Fortified Snack Reduced Anemia in Rural School-Aged Children of Haiti: A Cluster-Randomized, Controlled Trial
Source: PLoS One. 2016 Dec 22;11(12):e0168121. doi: 10.1371/journal.pone.0168121 (PMC5179061; doi:10.1371/journal.pone.0168121)
Supplement: S1 Protocol — (DOCX) [file pone.0168121.s002.docx]

***Vita Mamba* School Feeding Project**

**Study Protocol**

**Abstract**

This study builds on the *Mamba Lespri* School Feeding Project, conducted in Cap-Haitien, 2012-2013, to improve the nutrition of school-aged children, now in a rural context. Our main objective is to test the effect of a locally produced food, Vita Mamba, on reducing anemia and improving growth in school-aged children. The study uses a longitudinal randomized cluster design with three schools assigned to two groups : Group 1: Control (deworming), Group 2: Vita Mamba (and deworming). Schools will be matched on socio-economic, demographic, and nutritional characteristics and randomly assigned to one of the two comparison groups. Approximately 400 Haitian school children ages 3-16 years of age and their parents will be recruited and enrolled to be followed during the 2014-2015 school year. Data will be collected from two groups of participants at each school: children and parents. At baseline, socio-economic and demographic information will be collected from parents. Children will then be followed at two time points: at the beginning (baseline) and end (endline) of the study for nutrition measures (weight and height, and hemoglobin concentration). Parents will be surveyed for socio-economic characteristics, child diet and health, and anthropometry.

**Background and Significance.** The Haitian Ministry of Education has identified poor nutrition as a problem among Haitian school children. Poor nutrition stunts growth and development and compromises the immune system, leaving the body more vulnerable to disease. In Haiti, 20.9% of children less than five years are stunted and 11.4% are underweight (MSPP, 2013), but information about the nutritional status of the school-aged population is limited. One study of Haitian children between the ages of 11 and 19 years showed two-fifths were stunted (Psoter, et al, 2008).

The diets of school-aged children are likely deficient in vitamin A, iron, iodine, zinc, and potentially other micronutrients, similar to younger children in Haiti (Best, et al, 2010). Among 4-5 year olds, 35% were found to be vitamin A deficient and almost half were anemic (WHO, 2007), which can affect cognitive development, memory, and other mental function (NIH, 2011). Iodine deficiency, which has been associated with lower IQ scores, affects 59% of the entire Haitian population (MSPP, 2005). The exact prevalence of zinc deficiency in Haiti is unknown as is the case for most countries given the difficulties in measuring zinc nutrition though diarrhea prevalence, which is associated with zinc deficiency, is high in Haiti (MSPP, 2007). Intestinal parasites closely linked to nutrient deficiencies infect about a third of all Haitian school-aged children (WFP, 2010). Inadequate calories, protein and micronutrients stunt growth and intellectual development and compromise the immune system, leaving the body more vulnerable to disease (WHO, 2000).

In Haiti, despite the numerous and longstanding school feeding projects, there is limited information about the health and educational outcomes of these programs. Few programs have conducted evaluations designed to demonstrate impact or even program performance.  This study is designed to first characterize the nutritional situation of school-age children, and second, evaluate the effectiveness of a Ready-to-Use Supplementary Food (RUSF), Vita Mamba, for nutrition, health, and educational outcomes. It is among the first globally to examine a lipid-based RUSF in school-age children. The project will be implemented in the north of Haiti, in the Terrier Rouge zone. Vita Mamba will be introduced as a snack targeting children 3-12 years of age within a school feeding program.

**Study Aims:** To evaluate the nutrition impacts, especially growth and reduction of anemia, associated with the local product (Vita Mamba) offered through school feeding programs.

**Methodology**

**Study Design**. A quasi-experimental evaluation design is planned for this project.  Three primary schools will be matched on socio-economic and demographic factors and randomized into two participant groups:

*Group 1* (Control): Children 3-16 years of age in a school that does not normally participate in a school feeding program and will receive no food-based intervention during the study period. Deworming with Albendazole administered.

*Group* *2* (Vita Mamba): Children 3-16 years old in a school that does not normally participate in a school feeding program and who will receive Vita Mamba during the study period. Deworming with Albendazole administered.

**Study Activities**. This project will take place in schools in a communal section of the Terrier Rouge zone. This is a quasi-experimental study that will be coordinated by Washington University in St. Louis (WUSTL) in partnership with Meds & Food for Kids (MFK), Haiti’s National School Lunch Program (le Programme National de Cantine Scolaire, PNCS) and the Ministry of Public Health and Population (MSPP). There are three phases of the project: 1) planning, 2) intervention and 3) reporting and dissemination of findings.

Planning & Recruitment Phase. In the planning phase, there will be three primary activities: recruitment of staff and participants; training the study team; and program organization.

The Study Coordinator will be hired and begin work in September 2014. Two enumerators will be hired in October 2014. With the Principal Investigator, the coordinator will recruit and train the enumerators during the planning phase. The Study Coordinator will work with school personnel to enroll families of schoolchildren aged 3 to 16 years.

Parents/caregivers of eligible students will be acquainted with the study methods and procedures and will be asked to sign an informed consent form, which will be presented in the local languages (French and Creole). If parents/caregivers are unable to read, the consent form will be reviewed in full by French and Creole speaking trained enumerators to ascertain that the parent/caregiver has understood the nature of the study and is voluntarily enrolling their child in the study. A simple script will be reviewed with the students participating in the study to explain the RUSF product and the purpose of the anthropometric measurements and acceptability study.

Before the launch of the pilot study, WUSTL will form the study team, which will review and complete the necessary IRB certification program appropriate for this project. The training will include three (3) components: 1) Technical information and survey administration; 2) protocol instruction for anthropometric and hemoglobin concentration measure; and 3) field testing and validation exercises. The team of enumerators will receive detailed information regarding the product’s ingredients, functionality, and recommended consumption rates. Additional training will be provided to the team to explain the consent process as well as data collection and communication methodologies. Instructions will be given to the team on the correct methodology for conducting the acceptability study.

Intervention Phase.

**Sample Schools and Participants.** The intervention phase of the project will take place during the academic year from September 2014 through June 2015. Selection of the three schools in a communal section of the Great Basin region (Northeast Department) will be based on student socio-economic and demographic information, school size, and current school feeding program. All schools and students have an equitable chance of being included in the study. Three (3) schools will then be matched on important characteristics (basic anthropometry, SES, and school size) and will be assigned to one of two study groups described above: Group 1: Control (Danda); Group 2: Vita Mamba (Dolval).

At each of the three schools, two groups of participants will be part of the study: parents and children. A full description of the study will be provided and they will be invited to participate through the informed consent process. Parents and other caregivers of potentially eligible children will also be contacted at the beginning of the school year. They will be provided with a description of the study and invited to attend one of several meetings at the school with their children. At the meeting, the Study Coordinator will review and provide a full description of the study.

Children of interested parents/caregivers will be screened for eligibility based on the following criteria:

• Children 3-16 years old ;

• Children in good health [no fever, no congenital health condition];

• Children not severely malnourished [weight-for-height Z score >-2];

• Enrollment in the study school for the academic year 2014-2015.

Exclusion criteria are the following:

• Allergy to peanuts or soy;

• Fever or congenital or major health condition affecting the child;

• Child severely malnourished (weight-for-height Z score <-2).

Once eligibility is established, parents/caregivers will go through the informed consent process individually. The study staff will provide the children with a verbal description of the study, which will explain the purpose of the study, their role in the study and what to expect during the data collection methods. The children will be asked to give verbal assent. Following this, parents will be asked to return to the school on a scheduled date and the baseline survey will be administered.

**Sample size.** Power calculations were used to determine the adequacy of sample size, predetermined by total number of eligible children in the study schools and study budget. Using an estimate of difference-in-difference, mean change in Hb of 0.40 (SD 1.2) based on previous studies, adequate sample size need was estimated to be 143 per group [alpha=0.05 and power (1-β=0.80)] (Nga T. et al. 2009). With a design effect of 1.06 applied and an estimated 10% losses to follow-up, the study was found to be adequately powered to detect the hypothesized difference in Hb with 321 children enrolled (Wittes et al. 2002).

**Intervention & Methods.** Students in Group 2 will receive the RUSF/Vita Mamba snack daily for one academic year, 2014-2015. Each RUSF sachet (50g) contains 260 kcal and 100% RDA for most micronutrients. Simple messages about the importance of the RUSF, nutrition, and hygiene will be communicated at a low intensity level in group 2. All children will receive deworming, Albendazole, according to the national protocol of MSPP. Two types of surveys will be developed and administered for data collection in this study.

*Child measures and survey:* Students will be followed longitudinally during the school year on a range of health, nutrition, and education outcomes. A baseline, midline, and endline survey will be administered to assess nutrition and education impacts as well as acceptability of the product, Vita Mamba, to students as part of a school feeding program.

Hemoglobin concentrations in the students will be determined using the Hemocue system, which measures red blood cell status and is reported in grams per 100 milliliters (g/dl). A droplet of blood will be collected in a sterile fashion with a fingerstick and a microcuvette in one continuous process. The results will be compared to WHO anemia Hemoglobin cut-offs for children 5 - 11 years of age (WHO, 2011). The Seca Model 874 (Digital) 440 lb x 0.1 lb resolution and the ShorrBoard measuring board will be used to collect weight and height measures. These measures will be used to determine body mass index (BMI), anthropometric z-scores, and the prevalence of stunting, underweight and wasting, which will be compared to WHO Growth Standards.

*Parental survey:* At baseline and endline, a survey will be administered at the school to parents of children in the study. At baseline, after informed consent procedures, socio-economic and demographic information will be collected, including parental education, age, occupation, family size, income/assets, household water and sanitation services, health and nutrition behaviors, and access to healthcare. This will allow the evaluation team to control for confounding factors and stratify analyses. Further, additional recall data on frequency of consumption, dietary diversity, and morbidities of participating children will be collected from parents at both baseline and endline time points. Finally, height and weight measures will be taken on the parents.

Reporting & Dissemination Phase. During this phase, data will be analyzed and reports written based on the results of the study. Presentations will be made to donors, MSPP, PNCS, MFK, and the community with the results and next steps. Other dissemination activities could include writing policy and programming briefs and journal articles, and presenting at national and international conferences.

**Innovations and Impacts.** This is the first study to examine the effectiveness of a Ready-to-Use Supplementary Food (RUSF) with deworming in a rural Haitian context for addressing poor nutrition. If there are positive findings, the results may be used to increase and advance the school feeding programs in Haiti. Further, there is potential to affect policy and programming at the national level with regards to child nutrition in Haiti. At the international level, this study will contribute to the evidence base for supplementary foods as part of a school feeding protocol.

**Sources of Support**. This project is funded by the United States Department of Agriculture (USDA) Foreign Agricultural Service Micronutrient Fortified Food Aid Products program FFE-521-2012/034-00.

**Partners and Responsibilities**

Washington University in St. Louis (WUSTL)

Dr. Lora Iannotti, Principal Investigator

Sherlie Jean-Louis, Study Coordinator in Haiti

*This team works on the pilot study. Their tasks include development of the surveys, training of enumerators, collection of data, analysis of data, and reporting.

Ministry of Education/Haiti- Programme National de Cantine Scolaire (PNCS)

*This team is responsible for all programming activities of PNCS. In particular, their tasks include implementation of the school feeding programs.

Ministry of Public Health and Population/Haiti

* This team is responsible for ensuring the school feeding intervention is aligned with MSPP National Policy on Nutrition and with broader nutrition initiatives.

Meds & Food for Kids (MFK)

Dr Patricia Wolff, Executive Director

Jacques Raymond Delnatus
*This team organizes the logistics of distribution of the RUSF, Vita Mamba.

Appendix A: Risks and Benefits

Risks

There are less than minimal risks associated with this study.

While there could be concerns about allergic or other physical reactions by children to the peanut butter or soy, many studies in developing countries have found no such risk. There has been no indication of negative reactions to the food, allergy or otherwise, in other studies conducted around the world (Ghana, Malawi, Niger, etc.).  Infants and children in Haiti widely consume "mamba" or peanut butter without any adverse health consequences. A randomized controlled trial previously approved the National Bioethics Committee of Haiti (No 1011-23) and Human Research Protection Office (HRPO) of Washington University in St. Louis (IRB ID# 201101760) is testing the efficacy of a similar fortified peanut-butter paste, Nutributter. Among the 410 infants receiving this food, no peanut allergy has been identified.

In the Vita Mamba school feeding study, we will follow the same protocol to ensure there is a similar situation among school-age children in Haiti. When the fortified peanut-butter paste food is given for the first time, children will be monitored for any adverse reactions (itching, redness, rash, swelling, or difficulties in breathing) over a one-hour period. If a child reacts to the food, they will be treated immediately and become ineligible to continue in the study. For the remainder of the study, each school will be equipped with norepinephrine and diphendyramine (antihistamine) medication in case of allergic reactions at any time during the intervention period.

The measures of nutritional status are of minimal risk to the children. The finger prick associated with the Hemocue hemoglobin test will be no more painful than a vaccination shot, which children receive frequently. The study team will follow strict hygienic protocol with latex gloves and sterile equipment for the finger prick and handling of the samples.

Benefits

The direct benefits to the child will be decreased likelihood of nutrient deficiencies and improved health. Interventions that target prepubescent children have been shown to decrease the prevalence of nutritional deficiencies and improve school performance and overall child health (Soemantri, Pollitt, & Kim, 1985; Grantham-McGregor & Ani, 2001). Access to high quality, nutritious foods during the school day can boost enrollment, improve cognitive development, and increase performance measures on school exams (Soemantri, Pollitt, & Kim, 1985; Grantham-McGregor & Ani, 2001; Seshadri & Gopaldas, 1989). School feeding programs have also been shown to increase memory and the speed of processing by alleviating short-term hunger (Simeon & Grantham-McGregor, 1989).

The results of the study will demonstrate the effectiveness of using a lipid-based Ready-to-Use Supplementary Food (RUSF) as part of a school feeding program. While school feeding programs have been studied around the world, this study is among the first to examine a lipid-based RUSF in school-age children. It is intended to treat poor nutrition among school-age children on a population level with the possibility of long-term positive health and economic outcomes for the community. School feeding and increased enrollment have a positive influence on the economy and public health, which can be passed onto the next generation. The Government of Haiti is directly involved in this study and intends to use the results for future policy and programming in the country.

The new study in a rural area with the modifications described also allows the investigation of interventions to prevent anemia and promote growth with local products. The new study also allows us to test the RUSF against a strategy based on food.

Appendix B: Privacy and Confidentiality

Privacy

- During the recruitment process, the study team will visit with individuals at the time of school enrollment in a secure place at the school.
- During the consent process, the study team and participants will sit in a secure place such as an empty classroom at the school to ensure that they are appropriately distanced from others for privacy purposes.
- Each child will be measured at a reasonable distance away from the other children to ensure the privacy of the children. The three schools, which will be placed into one of two groups, have been selected so as to prevent contamination between the groups.
- Information collected will pertain to the well-being of the child as relates to the Vita Mamba, the food product. Any information beyond the scope of program evaluation and its acceptance will not be obtained.

Data Collection

- Paper/hard copy records (hard copy surveys, questionnaires, case report forms, pictures, etc.) – Surveys will be stored in a locked box at the Washington University work space in Haiti - Breda, Cap Haitien.
- Electronic records (computer files, electronic databases, etc.) - Only two computer hard drives will contain the names of participants. These computers will also be locked and secured in the Washington University work space in Haiti, Breda, Cap Haitien. Data files will be transferred periodically by jump drive back to the Brown School of Social Work to the computer hard drive of the PI of this study.
- The study team will use the Hemocue system for measuring hemoglobin samples. The disposable microcuvette, which is used to prick the child’s finger, collects a small amount of blood and then mixes it with the reagents immediately. The study team will then place the microcuvette into the portable analyzer, which will report the results on the display. After use, the microcuvettes will be placed in a standard, medical waste container and disposed of properly at the local health center.

Appendix C: Analysis

The primary outcomes tested by the study will be height, weight, and hemoglobin levels of the child, as markers of growth and micronutrient nutrition. The secondary outcomes include: nutrition (weight, diet, and dietary intake); health (morbidities such as diarrhea, and fever). Finally, the study will be examining the feasibility and acceptability of using Vita Mamba in the school feeding context and thus looking at ease of delivery.

The analysis methods will include descriptive and experimental data procedures. Several univariate analyses and multivariate regression analyses will be run to model outcomes and adjust for potential confounding factors. As a longitudinal study, the study will run panel regression by generalized least squared with random effects. Interaction terms will be introduced. As necessary, propensity score matching will be introduced to ensure the two groups are comparable.

**References**

Best C, Neufingerl N, van Geel L, van den Briel T, and Osendarp S. (2010) The nutritional status of school-aged children: Why should we care*? Food and Nutrition Bulletin. 31*(3), 400-417

Grantham-McGregor, S., &Ani.C. (2001).A Review of Studies on the Effect of Iron Deficiency on Cognitive Development in Children.Journal of Nutrition, 131(2), 649S–6S.

Menon P, Ruel MT, Loechl CU, et al. (2007) Micronutrient sprinkles reduce anemia among 9- to 24-moold children when delivered through an integrated health and nutrition program in rural Haiti. *J Nutr.,137*, 1023–30

Ministère de la Santé Publique et de la Population (MSPP), L'institut Haitien de l'Enfance. (2005) Fonds des Nations Unies pour l'Enfance. Enquete sur la prevalence de la carence en vitamine A et de la deficience end iode end Haiti.

Ministère de la Santé Publique et de la Population (MSPP). (2007) Haïti Enquête Mortalité, Morbidité et Utilisation des Services, EMMUS-IV, HAÏTI, 2005–2006, Institut Haïtien de l’Enfance (IHE). Pétion-Ville, Haïti, and Macro International Inc., Calverton, Maryland, USA.

Ministere de la Sante Publique et de la Population (MSPP), Institut Haitien de l’Enfance (IHE), ICF International. Enquête mortalité, morbidité et utilisation des services, Haiti, 2012. Calverton, MD: ICF International; 2013.

Nga TT, Winichagoon P, Dijkhuizen MA, et al. (2009) Multi- micronutrient-fortified biscuits decreased prevalence of anemia and improved micronutrient status and effectiveness of deworming in rural Vietnamese school children. *J Nutr., 139*:1013–1021.

Psoter, W., Spielman, Al, Gebrian, B., St. Jean, R., & Katz, R. (2008) Effect of childhood malnutrition on salivary flow and pH.*Arch Oral Biol, 53*(3), 231-7.

Seshadri, S., &Gopaldas, T. (1989). Impact of Iron Supplementation on Cognitive Functions in Preschool and School-Aged Children: The Indian Experience. American Journal of Clinical Nutrition, 50(3 Suppl), 675–84.

Simeon, D. T. & Grantham-McGregor, S. (1989) Effects of missing breakfast on the cognitive functions of school children of differing nutritional status. Am. J. Clin. Nutr., 49, 646-653.

Soemantri, A. G., Pollitt, E., & Kim, I. (1985.) Iron Deficiency Anaemia and Educational Achievement. *American Journal of Clinical Nutrition, 42*(6), 1221–8.

US National Library of Medicine, NIH.(2011) Iron deficiency anemia. Retrieved from [www.nhlbi.nih.gov/health/dci/Diseases/ida/ida_whatis.html](http://www.nhlbi.nih.gov/health/dci/Diseases/ida/ida_whatis.html)

vanStuijvenberg ME, Kvalsvig JD, Faber M, Kruger M, Kenoyer DG, Benadé AJ. (1999) Effect of iron-, iodine-, and beta- carotene-fortified biscuits on the micronutrient status of primary school children: a randomized controlled trial. *Am J ClinNutr, 69*, 497–503.

Wittes J. [Sample size calculations for randomized controlled trials.](https://www.ncbi.nlm.nih.gov/pubmed/12119854) Epidemiol Rev. 2002;24(1):39-53.

World Food Programme.(2010b). Overview. Retrieved from <http://www.wfp.org/countries/Haiti/Overview>

World Health Organization. (2000). Turning the tide of malnutrition: Responding to the challenge of the 21^st^ century. Geneva: WHO, 2000 (WHO/NHD/00.7)

World Health Organization.(2007). Vitamin and Mineral Nutrition Information System- WHO Global Database on Vitamin A and Anemia Haiti.

World Health Organization.(2011) Haemoglobin concentrations for the diagnosis of anaemia and assessment of severity.Vitamin and Mineral Nutrition Information System. Geneva, World Health Organization.

Wright, C., Sherriff A., Ward, S. McColl, H., Reilly J., & Ness, A. (2008).Development of bioelectrical impedance-derived indices of fat and fat-free mass for assessment of nutritional status in childhood.*Eur J Clin Nutr;62*(2):210-7.
